# Automated spheroid generation, drug application and efficacy screening using a deep learning classification: a feasibility study

**DOI:** 10.1038/s41598-020-67960-0

**Published:** 2020-07-06

**Authors:** Leo Benning, Andreas Peintner, Günter Finkenzeller, Lukas Peintner

**Affiliations:** 1 Department of Plastic and Hand Surgery, Faculty of Medicine, Medical Center, University of Freiburg, Freiburg, Germany; 2 Department of Computer Science, University of Innsbruck, Innsbruck, Austria; 3 Institute of Molecular Medicine and Cell Research, Albert Ludwigs University of Freiburg, Stefan Meier Strasse 17, 79104 Freiburg, Germany

**Keywords:** Biological techniques, Cytological techniques, Experimental organisms, High-throughput screening, Software

## Abstract

The last two decades saw the establishment of three-dimensional (3D) cell cultures as an acknowledged tool to investigate cell behaviour in a tissue-like environment. Cells growing in spheroids differentiate and develop different characteristics in comparison to their two-dimensionally grown counterparts and are hence seen to exhibit a more in vivo-like phenotype. However, generating, treating and analysing spheroids in high quantities remains labour intensive and therefore limits its applicability in drugs and compound research. Here we present a fully automated pipetting robot that is able to (a) seed hanging drops from single cell suspensions, (b) treat the spheroids formed in these hanging drops with drugs and (c) analyse the viability of the spheroids by an image-based deep learning based convolutional neuronal network (CNN). The model is trained to classify between ‘unaffected’, ‘mildly affected’ and ‘affected’ spheroids after drug exposure. All corresponding spheroids are initially analysed by viability flow cytometry analysis to build a labelled training set for the CNN to subsequently reduce the number of misclassifications. Hence, this approach allows to efficiently examine the efficacy of drug combinatorics or new compounds in 3D cell culture. Additionally, it may provide a valuable instrument to screen for new and individualized systemic therapeutic strategies in second and third line treatment of solid malignancies using patient derived primary cells.

## Introduction

Systemic therapies, i.e. chemotherapies and targeted-therapies, constitute one of the major pillars of modern cancer therapies and are of particular importance for the treatment of advanced and metastasized diseases. Today, the vast majority of all cancer patients dies due to metastases and their associated complications^1^. Despite the availability of evidence-based sequential or parallel therapeutic regimens for the most of all known cancer entities, physicians and patients alike regularly face the challenge of evolving resistance mechanisms that demand a robust fallback level. Once patients experience a disease progression under the respective first line treatment, they need to be administered a promising second or third line regimen. As guidelines tend to present treatments beyond the first or second line as equal options within the respective group, individual or institutional experiences drive the decision, if the enrolment in a suitable clinical trial is not an option. The search for a solution of this issue has contributed to pave the way for the field of personalized medicine over the past years, which focuses on the individual characteristics of a patient’s disease and custom tailors the best treatment option, respectively. The paramount interest in this field has just recently been underscored by the NIH’s Cancer Moonshot Blue Ribbon Panel that particularly emphasized the need for novel technologies to facilitate comprehensive cancer research with so-called human-derived next-generation cancer models^2^. The work presented in this article aims to contribute precisely to this proposed goal.

Major progress in the field of personalized medicine in cancer therapies was achieved through a better and more profound understanding of cancer itself: solid tumours are no longer seen as a collection of homogeneous malignant cells, but are rather considered a micro-evolutionary system that undergoes a constant transformation in response to endogenous and exogenous stimuli^3,4^. Several so-called lead mutations have been known for many years and are, in some cases, already important predictive indicators for a specific treatment. Nonetheless, these mutations only initiate the genomic dysregulation of malignant cells and are typically followed by further mutations. These so-called follow mutations contribute further to the genetic instability and diversity of a tumorous mass and eventually lead to the evolvement of numerous, genetically heterogeneous sub-entities^5^. Under a standard therapy, some of these sub-entities either develop mechanisms of resistance towards the applied agents or persevere better when nutrients and oxygen are at scarce—and hence gain dominance. Numerous approaches have been undertaken to approximate tumour response by modelling^6–8^. However, predictions about which sub-entities can persevere and the mechanisms they apply are, up to date, hard to make and can therefore not yet be taken into consideration when choosing the right regimen for an advanced cancer disease^9,10^. Just recently, Tuveson and Clevers presented a comprehensive review on cancer modelling by the means of organoid technologies and emphasized a number of key characteristics of three dimensionally grown cell cultures, namely the more accurate representation of interpatient variations, the ability to employ complex patient-derived cell sources and their capacity to report drug responses more reliably than conventional flat dish culture systems^11^.

In line with their promotion of sophisticated organoid models and a versatile usability thereof, several promising attempts on the generation of high-throughput screening assays for organoid cultures have been published. Yet, only few managed to integrate automation, size and spatial control, high numbers of replicates and a suitable interface for follow-up investigations so far. For example, Amann et al. demonstrated the generation of complex mono- or co-cultures. However, due to the complex generation mechanism discussed below, only low numbers of replicates were thoroughly examined by flow cytometry or microscopy^12^. Similar limitations apply to the work of Anastasov et al., who generated organoids similarly and focused on the detection of radiation resistant organoids and the effects of a simultaneous chemotherapy^13^. Other groups recognize scaffold-free 3D cultures as most suitable for mimicking in vivo phenotypes, but fall short of evaluating the tumour evolution^14^. Finally, some groups demonstrate a true high-throughput approach, but lack size and shape control to generate homogeneous organoids for follow-up investigations^15^. Although the results of this promising research as well as its limitations are clear indicators for the high complexity in this field, none of the approaches above incorporated a genuine computer-based readout for their experiments. From our perspective, this allows, with a reasonable effort, to reduce the complexity of a molecular or histochemical readout with comparable results.

Previous research in our group proposes an up-scale printing device that, in a next step, allows the simulation of the aforementioned micro-evolutionary process by generating a high number of homogenous cancer cell organoids from a tissue sample: organoids are generated by a printing platform employing a drop-on-demand (DOD) print head and are cultivated via the hanging-droplet method^16^. As using an automated drop-on-demand printing procedure increased the efficiency of spheroid production significantly, we now aim to apply evolutionary pressure, i.e. a specific cytostatic agent, a decrease in nutrients or pO2, to the spheroids and detect specific reactions of the individual spheroids. However, biochemical analysis remains labour intensive, as standard biochemical analysis methods demand a large quantity of source material. For instance, several hundred spheroids need to be pooled in order to yield a sufficient protein concentration to perform Western blot or qPCR analysis. Working on this problem, it became evident that spheroids undergo characteristic morphological changes when they are stressed, e.g. by cytotoxic agents. Images of individual spheroids can hence be analysed and classified by deep learning models^17^. Convolutional Neuronal Networks (CNN), if trained accordingly, allow to classify images in an efficient manner and are therefore predestined to assist—and eventually replace—costly and complex biochemical analysis. First introduced by LeCun and Bengio^18^, the application of CNN has enjoyed a rise of reputation in the past few years. In comparison to other neural networks, a CNN takes advantage of the spatial localisation inherent in images and hence identifies and extracts features.

The precise detection of morphologically aberrant spheroids and their subsequent analysis regarding cell viability and increase in cell death after exposure to cytotoxic agents by deep learning are at the focus of this paper. The project aims to provide a methodological feasibility study and therefore employs established and well characterized cell lines.

## Material and methods

### Cells and cell culture

All experiments were performed using immortalized adherent cell lines. HCT116, HEp2, HEK293T or mIMCD3 cells were maintained in standard cell culture vessels in DMEM medium supplemented with 10% FCS and 1% Pen/Strep at 37 °C in a 5% CO_2_ atmosphere. To maintain exponential growth, cells were split once they reached confluence, typically after 3 days. In order to grow the cells in spheroids, cells were washed with PBS, trypsinised with Trypsin/EDTA for 5–10 min and after all cells lost contact to the culture vessel, the reaction was stopped with the addition of an excess of DMEM. Cells were counted using a Neubauer counting chamber and diluted to a density of 1 × 10^6^ cells per ml.

### Automated spheroid production

**Figure 1:**
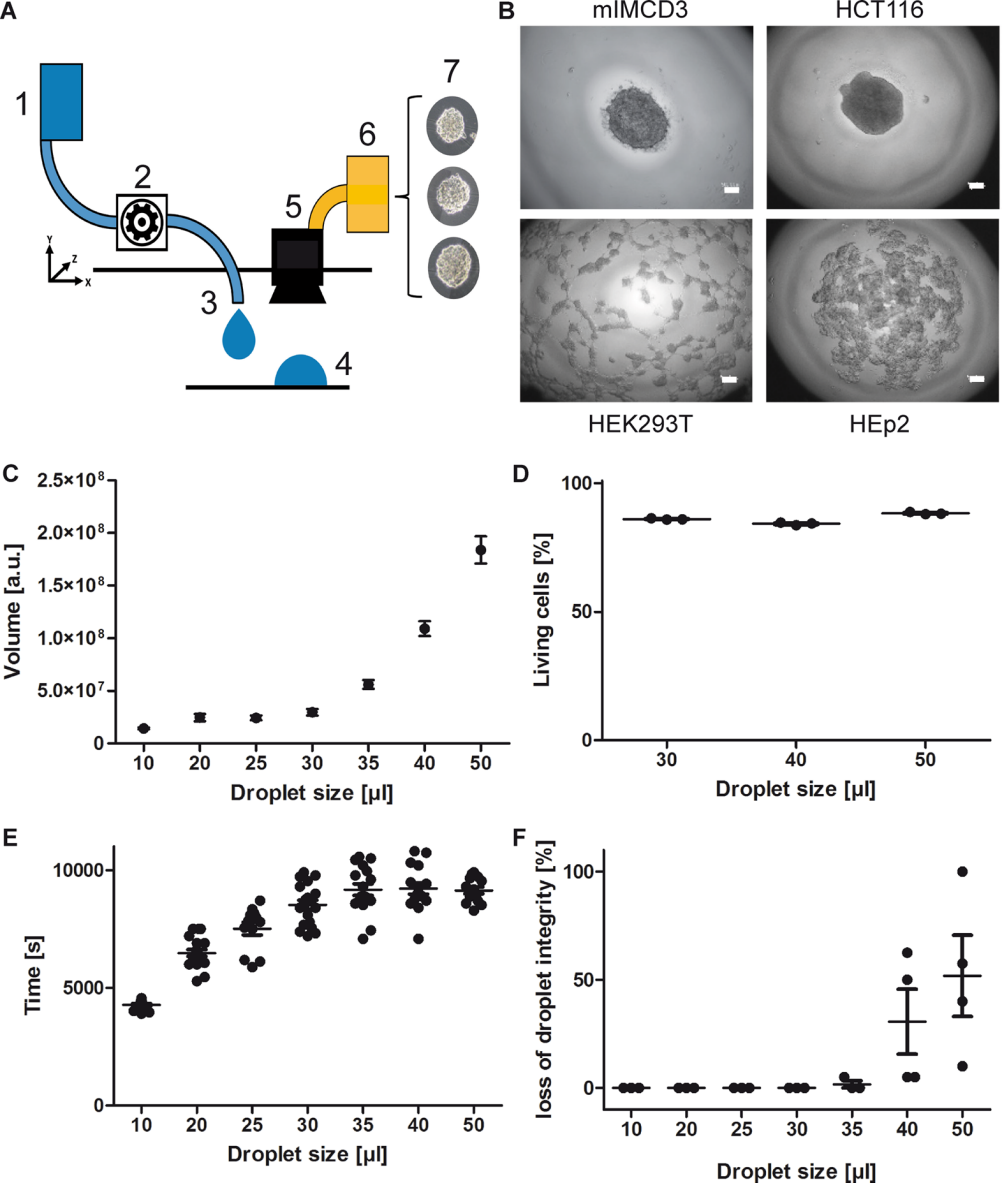
Spheroids form spontaneously in hanging drops. (**A**) Schematic representation of the spheroid seeding tool used throughout this work. Spheroids in hanging drops or cytotoxic drugs are seeded onto an inverted cell culture plate at various volumes using a peristaltic pump and a high precision x–y–z robot arm. A CCD camera monitors the spheroid quality and saves the images to a database. (1) Reservoir for cell suspension, (2) Peristaltic pump, (3) Nozzle for DOD droplet generation, (4) Droplet of homogenous shape and volume, (5) CCD camera, (6) Database, (7) Detection of shape and conformational alterations. (**B**) Representative images of spheroids of mIMCD3, HCT116, HEK293T or HEp2 cell lines forming spontaneously in 30 µl medium droplets containing 1 × 10^6^ cells/ml of trypsinised cells after 24 h. Size marker = 50 µm. (**C**) The volume of the spheroids consisting of mIMCD3 cells increases logarithmically depending on the droplet size/cell number. The volume was calculated measuring pixels of the captured images and is therefore set to arbitrary units [a.u.]. Cell concentration = 1 × 10^6^ cells/ml. Shown are means (horizontal line) ± s.d., n > 20. (**D**) Cell viability of mIMCD3 cells inside the spheroid after 48 h of incubation in spheroids of 30, 40 and 50 µl of volume. Cell viability was assessed by flow cytometry using Annexin-V counterstain. Cell concentration = 1 × 10^6^ cells/ml. Shown are individual survival rates (dots), means (horizontal line) ± s.d., n = 3. (**E**) Evaporation time of hanging drops (DMEM containing 10% FCS and 1% Pen/Strep) in a non-humid environment. Droplet sizes larger than 30 µl plateau at 8,500 s = 140 min. Shown are individual times (dots), means (horizontal line) ± s.d., n > 10. (**F**) The formation of hanging drops depends on an inversion of the carrier plate that holds the droplets. The larger the droplets are, the higher the probability that the droplets spontaneously trickle away during this process. Shown are percentages of spontaneously lost droplets on a plate bearing 80 droplets in three different experiments (dots), means (horizontal line) ± s.d., n = 3.

The DOD printing platform was developed on the basis of a computerized numerical control (CNC) milling machine (Sainsmart, Lexana, Kansas, USA), supplemented with an additive printer control chip (Anycubic, Frankfurt, Germany). The nozzle (200 µm size) was mounted to the x–y–z arm and connected to an infusion pump operated by a stepper motor, adjustable to the desired volumes after gravimetrical calibration. The nozzle aims at an inverted cell culture plate lid of 10 or 15 cm diameter that is placed 7 mm below the nozzle. A CCD camera (KKmoon, Shenzhen, China) is mounted next to the nozzle on the x–y–z arm. At 20 mm operation distance the camera captures images of individual spheroids and saves it to a database (Fig. 1a).

### Setup and process of spheroid production

Trypsinised floating cells at a concentration of 1 × 10^6^ cells per ml were loaded into the printing device. Droplets in the size of 30 µl were printed onto the inverted lid of a 15 cm cell culture plate in an array of 10 × 10 droplets. After seeding, the plate was carefully removed from the seeding device and quickly inverted. To avoid evaporation, the bottom of the 10 cm cell culture plate was filled with 10 ml PBS and the droplets bearing lid was put on the bottom plate. The spheroids were allowed to form in the droplets for 24 h in an incubator at 37 °C and 5% CO_2_. After 24 h the lid containing the spheroids was again loaded onto the seeding machine and 5 µl liquid containing 7 × concentrated drugs were applied into the droplets to yield a final 1 × concentration of drugs in the droplet containing the spheroid (35 µl final volume). Drugs used were Etoposide (ETO, final concentration: 20 and 100 mM) and Staurosporine (STS, final concentration: 2 and 20 µM). The lid bearing the droplets was again inverted and incubated for 24 h. To analyse the effect of the drugs on the spheroids, droplets were either scanned by the AI-connected camera or analysed by flow cytometry.

### Flow cytometry analysis

After imaging the spheroids, the lid containing the droplets was inverted, rinsed with 20 ml of PBS and all the spheroids were collected in a 50 ml tube. After centrifugation (5 min, 400 g), spheroids were trypsinised to obtain a single cell solution. After 5 min, DMEM + 10%FCS was added and cells were sedimented again. The pellet was resuspended in Annexin-V binding Buffer (10 mM Hepes, 140 mM NaCl, 2.5 mM CaCl_2_ in ddH_2_O) containing Annexin-V-APC (1:500). Cells were measured using a LSRII flow cytometer (Beckton Dickinson).

### Statistical analysis

Statistical analysis was performed where appropriate. Statistical significance was assessed by a two-way ANOVA and a Bonferroni post-test.

### Deep learning

The implementation is based on the python neural networks library Keras^19^. This library offers an ImageDataGenerator class that allows to generate batches of images with data augmentation. The following parameters for real-time data augmentation are used: rotation_range, rescale, shear_range, zoom_range, horizontal_flip. Designing the model, we focused on using a small convolutional network with few layers and a small amount of filters (neurons) per layer. The number of filters corresponds to the number of feature maps. A high dropout factor will regularize the model by randomly selecting neurons and ignore them during the training. This results in various independent representations and the model will incorporate these into its learning process to avoid overfitting^20^. Specifically, we trained a convolutional network with 4 convolutional layers. Each layer has a rectified linear unit (ReLU) activation function, which projects negative weights to zero, and is followed by max-pooling layers with a pool size of 2 × 2. Pooling is needed to eliminate redundant information and speed up training in addition to ReLU and dropout. After the fourth pooling layer, activations were flattened and two subsequent layers with 128 and 3 features were fully connected. The model ends with a softmax activation to produce a probability distribution over given classes. The categorical model summary can be seen in Table 1 and has overall 6,913,091 trainable parameters.

**Table 1:** Categorical model summary.

Layer (type)	Output shape	Parameter #
conv2d_1 (Conv2D)	*(None, 398, 318, 32)*	320
activation_1 (Activation)	*(None, 398, 318, 32)*	0
max_pooling2d_1 (MaxPooling2D)	*(None, 199, 159, 32)*	0
conv2d_2 (Conv2D)	*(None, 197, 157, 64)*	18,496
activation_2 (Activation)	*(None, 197, 157, 64)*	0
max_pooling2d_2 (MaxPooling2D)	*(None, 98, 78, 64)*	0
conv2d_3 (Conv2D)	*(None, 96, 76, 64)*	36,928
activation_3 (Activation)	*(None, 96, 76, 64)*	0
max_pooling2d_3 (MaxPooling2D)	*(None, 48, 38, 64)*	0
conv2d_4 (Conv2D)	*(None, 46, 36, 128)*	73,856
activation_4 (Activation)	*(None, 46, 36, 128)*	0
max_pooling2d_4 (MaxPooling2D)	*(None, 23, 18, 128)*	0
flatten_1 (Flatten)	*(None, 52,992)*	0
dense_1 (Dense)	*(None, 128)*	6,783,104
activation_4 (Activation)	*(None, 128)*	0
dropout_1 (Dropout)	*(None, 128)*	0
dense_2 (Dense)	*(None, 3)*	387
activation_5 (Activation)	*(None, 3)*	0

The complete source code is uploaded to GitHub and can be accessed upon reasonable request.

## Results

We aimed to develop a tool that allows the generation of spheroids in high quantities and in a constant quality with regard to spatial control and size distribution. Furthermore, it needed to be able to treat the generated spheroids with preselected drugs and should be able to capture each individual spheroid in a hanging drop with a CCD camera. The resulting tool consists of an x–y–z platform that holds a peristaltic pump, which applies defined amounts of liquid containing trypsinised epithelial cells in a defined pattern on the lid of a cell culture plate (Fig. 1a). The droplet sizes generated by the infusion pump remained constant with only very small standard deviations (Supplementary Fig. 1). After applying the cells in a specific grid-pattern, the cell culture plate is carefully inverted and incubated for 24 h in a standard cell culture incubator. Next, the plate is inverted again and placed into the printing device. Once the pre-formed pattern is automatically identified, therapeutics in different concentrations are infused into the existing droplets via DOD printing, as outlined above. After a 24-h incubation period, the effect of the drugs is assessed by a CCD camera that identifies the individual spheroids and compares the condition of each separate spheroid to a database of previously diagnosed, i.e. labelled, spheroids.

### Tumour spheroids form spontaneously in hanging drops

As shown in various previous studies, cells from diverse sources will form spheroids spontaneously when grown in an environment where cells can only attach to each other, as it happens in hanging drops^21,22^. However, depending on the cell line or differentiation status, cells can aggregate and form a ball shaped single spheroid (Fig. 1b, mIMCD3 and HCT116), or form several interconnected small spheroids on the bottom of the droplet (Fig. 1b, HKT293T and HEp2).

The volume of the spheroids grows logarithmically in relation to a linear increase in the cell number contributing to the spheroid (Fig. 1c, mIMCD3 cells). The formation of a spheroid rapidly establishes a supply gradient that exposes the inner cell mass to a gradually increasing scarcity in nutrients and oxygen. However, the increase in cell number included into the spheroid does not lead to an increase in spontaneous apoptosis after 48 h in culture, averaging around 80% living cells in spheroids larger than 60.000 cells (Fig. 1d). Nonetheless, too small droplets bear the risk of rapid evaporation of the medium before the spheroids can be placed in the incubator (Fig. 1e) and too large droplets, containing more than 35 µl (or 35.000 cells), cannot be inverted securely without the danger of the droplets of flowing uncontrollably on the carrier material and losing its pre-formed grid-pattern (Fig. 1f).

Based on this finding we chose a concentration of 1 × 10^6^ cells/ml as an optimal medium-to-cell ratio when growing spheroids in further experiments. Droplet sizes of 30 µl proved to be the ideal volume to form spheroids since handling and efficacy demonstrated to be optimal in this setting (Fig. 1c, e, f and Supplementary Fig. 1).

### Spheroids react to drug exposure

**Figure 2:**
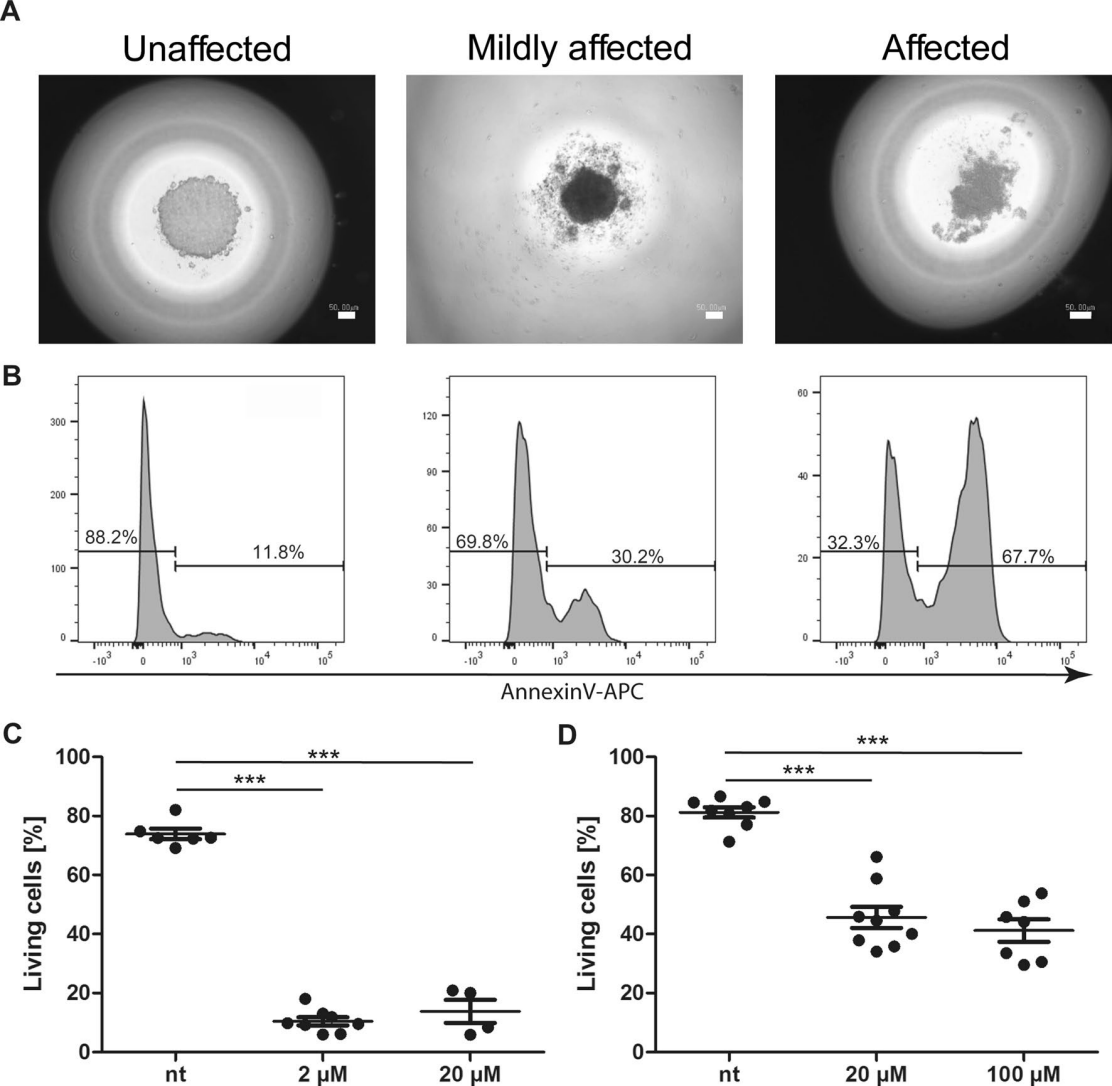
Spheroid integrity is affected by the addition of cytotoxic drugs. (**A**) Representative images of spheroids consisting of mIMCD3 cells that are either labelled ‘unaffected’ (left image, > 80% living cells), ‘mildly affected’ (middle image, 40–60% viability) or ‘affected’ (right image, < 40% viability. Size marker = 50 µm. (**B**) Corresponding flow cytometry analysis to images presented in (**A**). Cell viability was assessed by flow cytometry using Annexin-V counterstain. (**C**) Viability of spheroids consisting of mIMCD3 cells exposed for 24 h to the pan kinase inhibitor Staurosporine in the indicated concentrations. N = 4–9, *p* < 0.001 for nt against both treated conditions. (**D**) Viability of spheroids consisting of mIMCD3 cells exposed for 24 h to the topoisomerase inhibitor Etoposide in the indicated concentrations. N = 7–9, p < 0.001 for nt against both treated conditions.

Cells in hanging drops start to form spheroids within minutes after seeding. After 24 h under suitable incubation conditions, the spheroids of mIMCD3 or HCT116 cells condense to a stable cell mass (Fig. 1b). These matured spheroids can then be treated with cytotoxic agents. Directly applying the agent into the liquid of the hanging drop enables a penetration of the agent into the spheroid and exposes the cells to its biological effect. Depending on the sensitivity of the cells towards the cytotoxic agent, cells exhibit clear signs of cytotoxicity in a time and dose dependent manner: apoptotic cells within the spheroid disturb the cohesion of the cells and cause a shift from high contrast edges towards an irregular appearance. This finding was particularly prominent in mIMCD3 spheroids after 24 h of incubation (Fig. 2a), but was also seen in other spheroid-forming epithelial cell lines. The association of the altered morphology and the viability of the spheroids was evaluated via flow cytometry (Fig. 2b). Applying the pan kinase inhibitor Staurosporin to the spheroids induces massive cell death within the spheroid after 24 h of incubation (Fig. 2c). The topoisomerase inhibitor Etoposide, however, only induces mild cell death in spheroids, even despite high concentrations (Fig. 2d), probably because cells in spheroids do not actively enter the cell cycle anymore and therefore are not specifically sensitive to DNA damage^23^. The same cells grown in standard 2D cell culture conditions still show high sensitivity towards Etoposide exposure (Supplementary Fig. 2).

### Neural networks are able to discriminate between drug sensitive and non-sensitive spheroids

**Figure 3:**
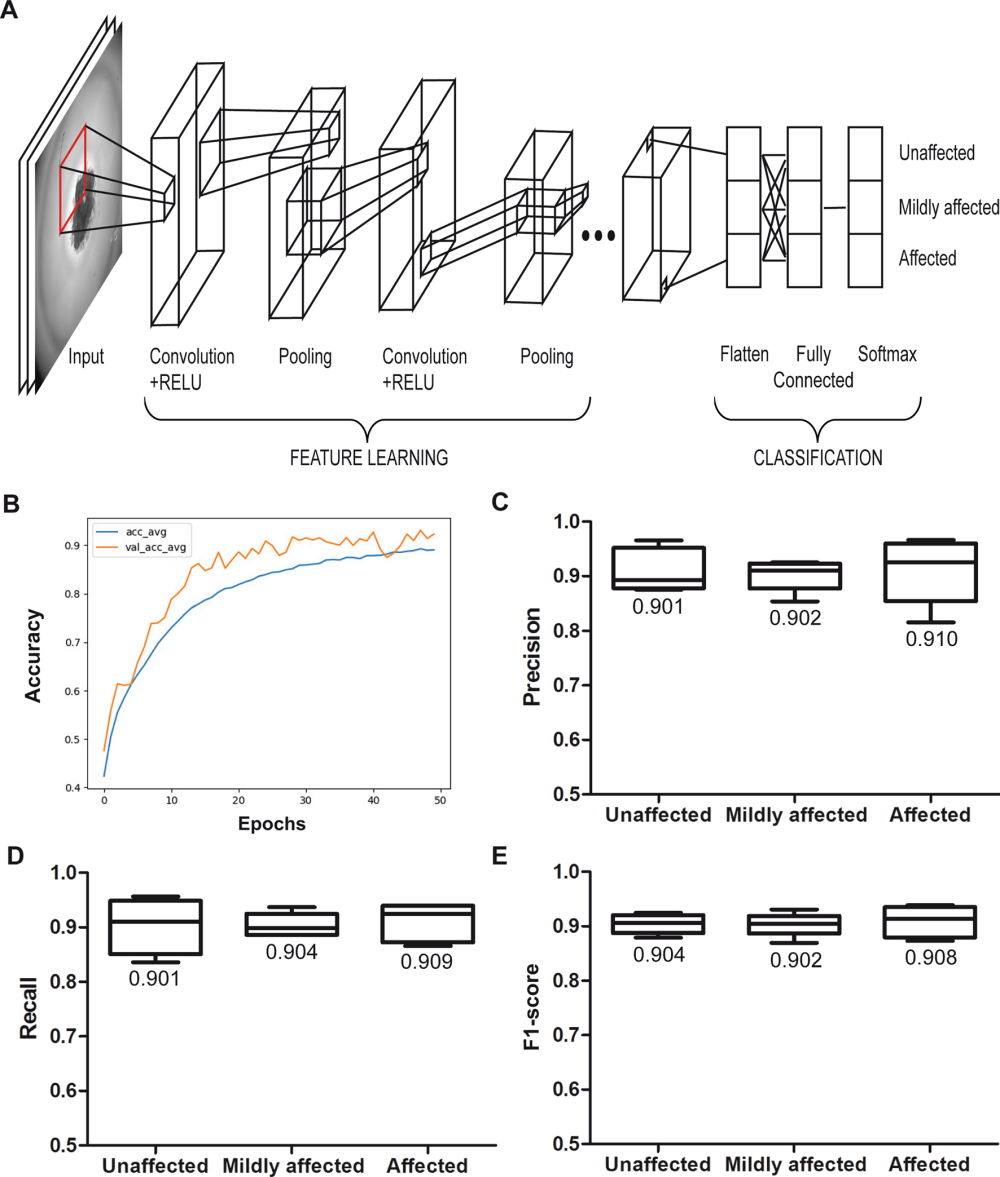
Neural Networks are able to discriminate between drug sensitive and non-sensitive spheroids. (**A**) Graphical representation of the convolutional neural network (CNN). Images are convoluted with kernel matrices in order to reduce complexity but to keep the image information. Fully connected layers are then used to annotate an input image to a given classification. (**B**) Averaged training accuracy (‘acc_avg’, blue line) and validation accuracy (‘val_acc_avg’, orange line) of the CNN in a five-fold cross validation for 50 epochs. (**C**) The level of precision of the image classification by the CNN as calculated by a five-fold cross validation for the three individual categories “unaffected”, “mildly affected” and “affected. Numerical values are the means. (**D**) The level of recall of the image classification by the CNN as calculated by a five-fold cross validation for the three individual categories “unaffected”, “mildly affected” and “affected. Numerical values are the means. (**E**) The F1-score as a function from precision (**C**) and recall (**D**) for the three individual categories of spheroid viability: F1 = 2 × (precision × recall)/(precision + recall). Numerical values are the means.

The analysis of spheroid sensitivity towards cytotoxic agents, as presented in Fig. 2, is very cost and work intensive and a pool of > 100 equally treated spheroids had to be measured by flow cytometry to yield reasonable cell numbers. To circumvent this detriment, we trained a deep learning model to estimate the sensitivity of spheroids toward a given drug. All spheroids that were analysed in Fig. 2 were individually micrographed before disassembling the spheroids for flow cytometry analysis. After determining the viability of the spheroids by flow cytometry, the corresponding images were grouped into three categories: ‘unaffected’ (> 80% viability), ‘mildly affected’ (40–60% viability) and ‘affected’ spheroids (< 40% viability). This set of categorised images was then used to train a deep learning model based on an AlexNet CNN architecture (Fig. 3a). For our model different CNN architectures (DenseNet, VGG, ResNet^24–26^) were tested, but did not delivered satisfactory results. AlexNet performed best, marked a starting point for our architecture and resulted in the described model^27^. 400 images per category were generated for the training and test phase, leading to a total of 1,200 images. To evaluate the model five-fold cross validation was used. We split our data into 80% training and 20% test set first. Regarding to fivefold cross validation, this training set was then used to create 5 splits of training and validation sets. Averaged training accuracy and validation accuracy over all folds are presented in Fig. 3b. After 50 epochs, our model reached in average a training accuracy of 0.89 and a validation accuracy of 0.92 (Fig. 3b). The best performing model was then used on the separate test set and predicted all three categories with a precision of about 0.9 (Fig. 3c), where 1.0 could be considered as the perfect result with 100% correct classifications. Relevant designations were identified by the recall measure and yielded values for all three categories higher than 0.9 (Fig. 3d), which further proves the efficacy of the model. A harmonic mean between precision and recall results into the F1-score (Fig. 3e) and further supports the findings above: for all three categories the mean of the F1-score is around 0.9. However, the standard deviation seems to increase for spheroids classified as ‘affected’. The cross validation demonstrates that the CNN is able to discriminate visual images between untreated spheroids and spheroids that are exposed to chemical compounds that induce cell death.

## Discussion

The last two decades brought the insight, that many cells show entirely different physiological behaviours when they grow in a 3D culture environment^28,29^. This effect may constitute one of the reasons, why so many promising new drug candidates fail in the clinical application^30^. To tackle this issue, testing new compounds on three dimensionally grown spheroids or organoids is more and more turning into the new standard of drug research^30,31^. However, efficient production and handling is a major issue of spheroid research and numbers for statistical analysis remain notoriously low^32^. Several commercial applications offer tailored kits that aim to address this issue, but fail to fully overcome the current limitations, as discussed in the introductory paragraph. Particularly, the reliance on multi-component commercial kits that require a repeated transfer of cells and spheroids^12^, the limited variation of the experimental setup and a low spatial control of spontaneously aggregated spheroids are of major concern. Furthermore, we consider the frequent use of Matrigel as a structural supplement as noteworthy^15^, since it is derived from the murine Engelbreth-Holm-Swarm Sarkoma. Although it provides a widely accepted and particularly in vivo-like model for extracellular matrix (ECM), it has ever since lacked a precise classification of its components and characteristics^33,34^. It therefore accounts for a high level of heterogeneity in any model for 3D cell culture. We are well aware that heterogeneity is a crucial characteristic of any tumour ECM—which is not yet sufficiently reflected by our model -, but consider this as a particular challenge for deep learning based classifications that needs to be addressed by further research. Here, we focus on a new approach on generating, treating and analysing spheroids with the perspective of providing supplementary information for patient specific therapy decisions. The design of an automated x–y–z pipetting device allows (a) efficient mass production of hanging drops on the lids of 15 cm cell culture dishes, (b) efficient treatment of hanging drops with drugs and (c) rapid analysis of spheroid viability by an image-based deep learning algorithm. While the automated seeding of spheroids did not yield significant time savings compared to manual spheroid production, the precise application of drugs into the existing hanging drops was much faster and more accurate than the manual approach. Generating vast quantities of spheroids and treating them with drugs for further biochemical analysis becomes hence much more time and resource efficient.

Feeding the CCD-generated close caption images to a deep learning model enabled a classification of the spheroids. Treatment of spheroids with cytotoxic agents activates cell stress responses and ultimately the cells in the spheroid enter apoptosis. Up to date, the gold standard methods to measure cell death are usually either protein based or single cell based, but these methods demand a high number of cells that cannot be provided by a single spheroid. We therefore used the flow cytometry based Annexin V staining on phosphatidylserine as a criterion standard^35^ on a pool of 80 identically treated spheroids. Before the biochemical analysis was performed, all 80 spheroids were imaged and later tagged according to the survival rate measured by flow cytometry. These photos and the respective tags were then employed to train the deep learning model based on a CNN, as it has been presented for other diagnostic issues with comparable results^36,37^. We are aware that our set of labelled images is relatively small in comparison to other datasets used to train neural networks. We hence aim to further underline the capacity of our model with independent training and validation sets. Yet, for the here presented proof of concept, we consider a cross validation a suitable and reasonable approach to demonstrate the feasibility of our setup. Furthermore, the limited number of images and the requirement of our setup to work robustly on these data sets represents the general setting we expect for the envisaged clinical application of the presented approach. It is, nonetheless, noteworthy that small datasets consequently lead to the problem of overfitting. An overfitted model is not able to generalize, i.e. yielding a high accuracy at classification with the data used in the training period, but with a significantly lower accuracy when new test data is used. To avoid overfitting, we employed measures that can help to improve a model, like data augmentation or the entropic capacity of a model. Entropic capacity represents the amount of information a model will store. The less features a model can store, the more it will focus on the most significant features. To address these issues, we designed a model with few layers and filters. Furthermore, images were manipulated via data augmentation. However, as outlined above, we aim to increase the number of labelled images used for training to improve the overall generalization capabilities of our model. Additionally, the application of more complex neural networks designs, like the competition-winning architecture ResNet^24^ or VGG^25^, would be facilitated through more available training data. Besides increasing the learning data set, we also aim to use a pretrained model, which already has learned features that are relevant for our specific classification problem of spheroids or organoids. Another promising approach would be the use of a generative adversarial network (GAN). This system consists of two networks that challenge each other. The generative network (generator) generates image candidates and tries to trick the discriminative network (discriminator), which aims to differ between real and generated images^38^. Hence, our model could serve as an initial discriminator in a GAN and is at the focus of a future project.

Although methodologically different, we consider our project as a complement to the previous work of other groups, which have achieved significant progress in the field of high throughput 3D culture screening. Particularly, we aim to discuss the compatibility of the different approaches to define further fields of study. Celli et al. presented an interesting work flow for the evaluation of 3D tumour models, but relied on pre-defined parameters for their quantitative readout^39^. From our experience, the incorporation of calcein and ethidium bromide as well as trypan blue is difficult to establish on 3D cultures, since all agents tend to distribute unevenly. This phenomenon has already been described before^40^ and is even more relevant when Matrigel-embedded heterogeneous 3D cultures are employed, as this group reports. Although the latter aspect mimics an in vivo situation better than homogeneously distributed 3D cultures, as highlighted above, it complicates the characterization of the organoids on the basis of a deep learning classification enormously. Furthermore, our setup is by now not able to detect fluorescent signals and can hence not apply a respective algorithm to detect the distribution of fluorescent dyes^39^. For future works, we will evaluate the validity of fluorescent markers in 3D cultures, but tend to incorporate metabolic sensors for a more specific analysis. Another promising approach has been presented by Zanoni et al., who put a particular focus on the spherical configuration and volume of the 3D cultures. However, our findings do not support their proposed theory that more irregularly shaped spheroids yield a higher viability than spherical ones^41^. Yet, we consider larger 3D cultures with diameters of 800–900 µm a suitable option to increase the in vivo similarity of our approach, but failed to generate spheroids of this size by the hanging droplet method, as outlined above. Again, our experience is that trypan blue exclusion is not a reliable parameter for 3D cultures and, although thoroughly investigated by this group, bioluminescence based assays neither seem to penetrate complex large 3D culture systems sufficiently. Other groups propose intracellular stains, i.e. calcein AM and ethidium bromide or familiar substances, as a suitable qualitative marker, but do not extend their use to quantitative comparisons^42,43^. In general, we question that simple morphological parameters, i.e. diameter, perimeter, volume, surface area and sphericity are suited as individual markers for 3D culture screening, as proposed by various groups^44–46^. However, an integrated deep learning approach, as presented in this article, allows the incorporation of these and many more parameters into the classification process. A very sophisticated approach towards the screening of 3D cultures was presented by Bulin et al., which included a stromal component into the setup. In order to mimic in vivo-like circumstances, we consider this an essential step and aim to incorporate stromal cells into our screening approach in future works, as our homogeneous 3D cultures without a suitable ECM do not yet mimic an in vivo environment to a sufficient degree. However, although complex, we consider the presented multi-step workflow as error-prone, since (a) conventional fluorescent stains are once again used as a quantitative readout and (b) the analysis is essentially based on two pre-defined parameters^47^. Nonetheless, we understand the presented platform as a robust tool for the screening of complex and structurally heterogeneous 3D cultures and see an opportunity to increase the versatility of our deep learning classification to more complex organoids.

**Figure 4:**
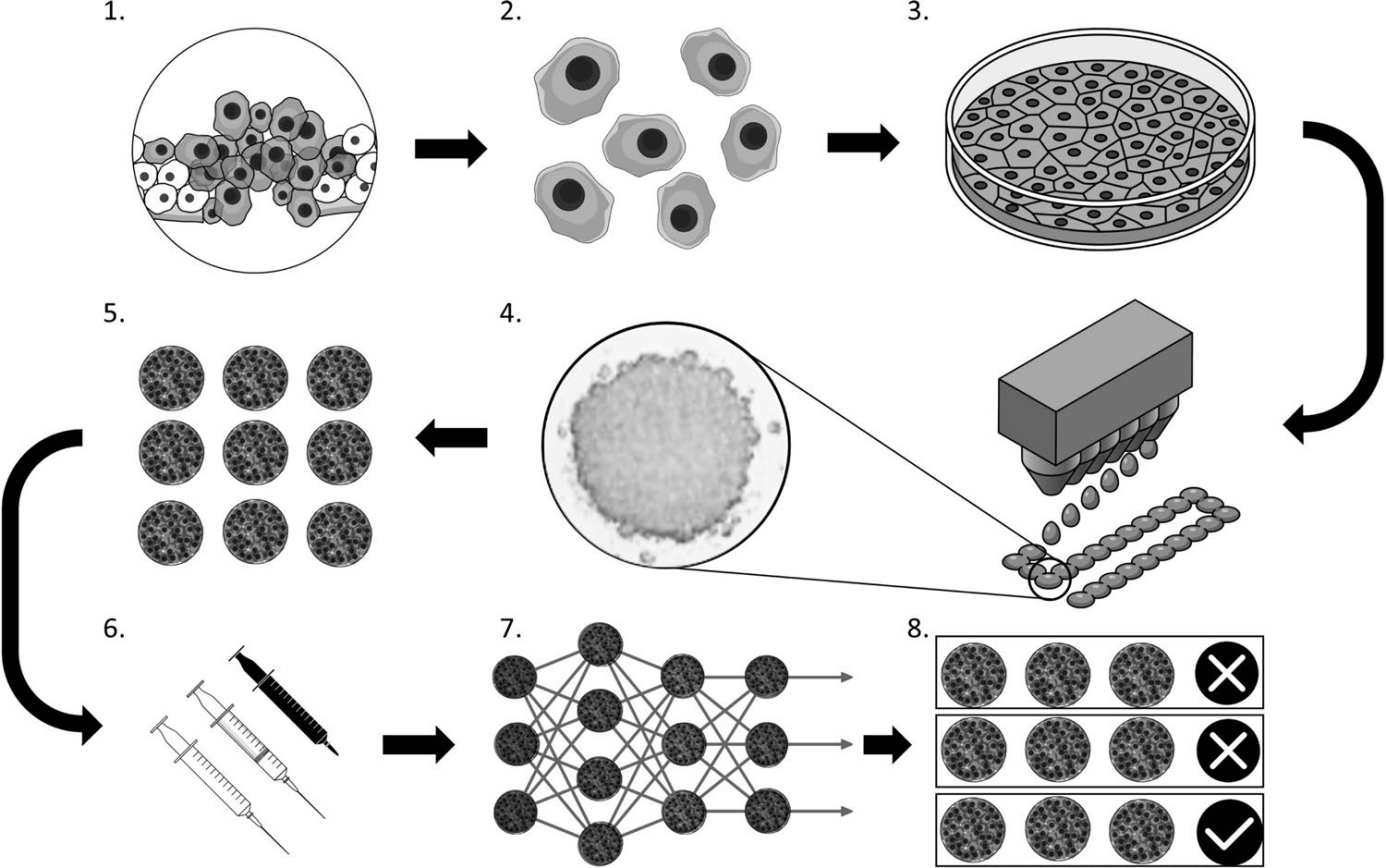
Proposed workflow to optimize drug screening in solid tumours. (1) A malignant lesion grows in vivo. (2) Primary cells are secured by a biopsy and (3) expanded to yield a sufficient number of cells in vitro. (4) Homogeneous spheroids are generated via the hanging drop method and (5) are incubated until spheroids are fully matured. (6) Combinations of different drugs and concentrations are applied and the response of each spheroid is tracked via imaging. (7) Images are analysed by a pre-trained CNN and (8) allow a classification regarding the sensitivity towards a specific drug or concentration.

The promise of personalized medicine rests on the tailored treatment of a patient with a therapeutic regime that demands efficient determination of drug-disease-interaction for each individual patient. Our approach promises an easy to operate all-in-one tool that combines the strength of two innovative concepts—in vivo-like 3D cell culture and a deep learning environment, to enable personalized medicine in clinical applications (Fig. 4). By establishing a reliable automated screening approach for spheroids and their respective responses to the administered therapeutic agents or environmental parameters, we aim to detect mutation tendencies within the primary tissue samples that, as a long term goal, could support the decision making process for specific and personalized treatment regimens.

## Supplementary information

Supplementary information

## Abbreviations

CNN: Convolutional neural network
DOD: Drop on demand
ECM: Extracellular matrix
GAN: Generative adversarial network
2D: Standard two-dimensional cell culture
3D: Three-dimensionally grown spheroids in cell culture
