# Supplementary material for: Automated spheroid generation, drug application and efficacy screening using a deep learning classification: a feasibility study
Source: Sci Rep. 2020 Jul 6;10:11071. doi: 10.1038/s41598-020-67960-0 (PMC7338379; doi:10.1038/s41598-020-67960-0)
Supplement: Supplementary file 1 — Supplementary information [file 41598_2020_67960_MOESM1_ESM.pdf]

Supplementary Information

Automated spheroid generation, drug application and efficacy screening using a deep learning classification: A feasibility study

Leo Benning[1,4], Andreas Peintner[2,4], Günter Finkenzeller[1] and Lukas Peintner[3,*]

[1]Department of Plastic and Hand Surgery, Faculty of Medicine, Medical Center, University of Freiburg, Freiburg, Germany

[2]Department of Computer Science, University of Innsbruck, Innsbruck, Austria

[3]Institute of Molecular Medicine and Cell Research, Albert Ludwigs University of Freiburg, Germany

[4]Contributed equally to this work

*Corresponding author: L. Peintner, Institute of Molecular Medicine and Cell Research, Albert Ludwigs University of Freiburg, Stefan Meier Strasse 17, D-79104, Freiburg, Germany, Tel: +49 761 203 9618; Fax: +49 761 203 9620; E-mail: lukas.peintner@mol-med.uni-freiburg.de

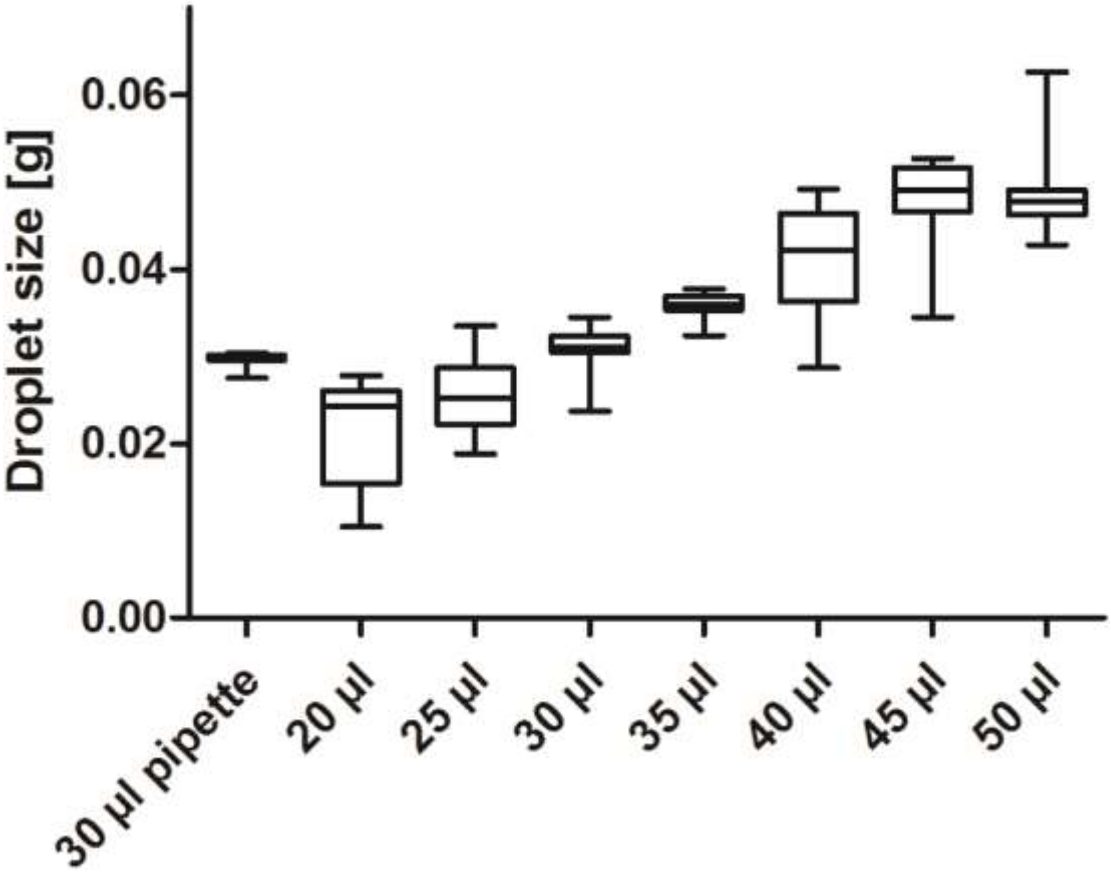

Supplementary Figure 1: High throughput spheroid generation and screening.

Hanging drops can be mass produced by a fully automated pipetting robot. Droplets produced by the pipetting robots are very homogeneous and are matching to manually produced ones (30 µl, pipette). Droplet volume was assessed by gravimetrical analysis of the individual droplets. N < 20.

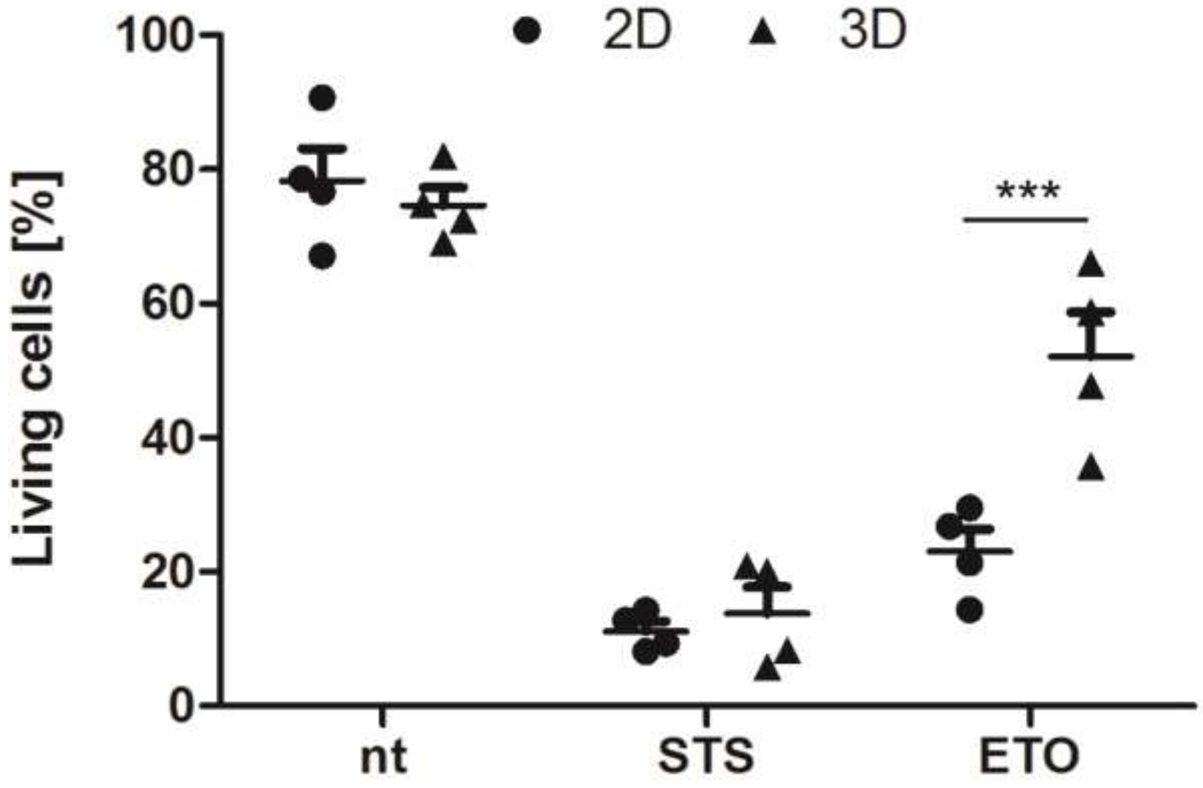

Supplementary Figure 2: Cells change their sensitivity to drugs depending on their growth situation.

mIMCD3 cells grown in 2D or 3D cell culture for 24 hours and then treated with Staurosporine (STS, 2 µM) or Etoposide (ETO, 20 mM) for 24 h. Cell viability was assessed by flow cytometry. N = 4, $p<0.001$.
